# Development of Sustained-Release Ophthalmic Formulation Based on Tranilast Solid Nanoparticles

**DOI:** 10.3390/ma13071675

**Published:** 2020-04-03

**Authors:** Misa Minami, Ryotaro Seiriki, Hiroko Otake, Yosuke Nakazawa, Kazutaka Kanai, Tadatoshi Tanino, Noriaki Nagai

**Affiliations:** 1 Faculty of Pharmacy, Kindai University, 3-4-1 Kowakae, Higashi-Osaka, Osaka 577-8502, Japan; 1611710015s@kindai.ac.jp (M.M.); 1611610157u@kindai.ac.jp (R.S.); hotake@phar.kindai.ac.jp (H.O.); 2 Faculty of Pharmacy, Keio University, 1-5-30 Shibakoen, Minato-ku, Tokyo 105-8512, Japan; nakazawa-ys@pha.keio.ac.jp; 3 Department of Small Animal Internal Medicine, School of Veterinary Medicine, University of Kitasato, Towada, Aomori 034-8628, Japan; kanai@vmas.kitasato-u.ac.jp; 4 Faculty of Pharmaceutical Sciences, Tokushima Bunri University, 180 Yamashiro-Cho, Tokushima 770-8514, Japan; tanino@ph.bunri-u.ac.jp

**Keywords:** tranilast, sustained delivery system, lacrimal fluid, eyelid, meibomian glands

## Abstract

Eye drops containing Tranilast (TL), N-(3,4-dimethoxycinnamoyl) anthramilic acid, are used as an anti-allergic conjunctivitis drug in the ophthalmic field. Traditional eye drops are very patient compliant, although the bioavailability (*BA*) of most eye drops is low since eye drops cannot be instilled beyond the capacity of the conjunctival sac due to its limited volume. Thus, traditional eye drops have low *BA* and a short duration of the drug on the ocular surface, so solutions to these problems are highly anticipated. In this study, we designed a sustained-release drug-delivery system (DDS) for TL nanoparticles. TL nanoparticles were prepared by bead mill treatment, and the gel formulations containing TL nanoparticles (TL-NPs-Gel, particle size 50 nm–100 nm) were provided by carboxypolymethylene. The crystal structure of TL with and without bead mill treatment is the same, but the TL solubility in formulations containing nanoparticles was 5.3-fold higher compared with gel formulations containing TL microparticles (TL-MPs-Gel). The photo and thermal stabilities of TL-NPs-Gel are also higher than those of dissolved TL. Moreover, when TL-NPs-Gel is applied to the upper eyelid skin (outside), the TL is released as nanoparticles, and delivered to the lacrimal fluid through the meibomian glands. In addition, the TL release profile for TL-NPs-Gel was sustained over 180 min after the treatment. These findings can be used to develop a sustained-release DDS in the ophthalmic field.

## 1. Introduction

Eye drops are considered to be the topical delivery route of drug administration for the treatment of ophthalmic diseases and are extensively utilized as pharmaceutical formulations [1]. Although this route is the most patient compliant, the rapid physiological drainage of the tear film results in low retention on the ocular surface. In addition, the bioavailability (*BA*) for most eye drops is low, since the eye drops cannot be instilled beyond the capacity of the conjunctival sac due to its limited volume, and ocular functions, such as blinking, nasolacrimal drainage and tear turnover, also limit the *BA* of drugs instilled as eye drops [2,3]. Moreover, eye drops do not allow for drug instillation during sleep. Thus, drugs delivered in traditional eye drops have low *BA* and a short duration on the ocular surface. Solutions to these problems are expected.

The eyelid is the thin skin located on the ocular surface with the thickness on the human body <1 mm [4]. There are some reports to target the eyelid, and Kimura and Tojo [5] and Isowaki et al. [6] investigated drug levels in the conjunctiva in an in vivo study involving transdermal delivery through the eyelid. In addition, See et al. reported on drug delivery within the ocular region through the lower eyelid skin using hydrophobic drugs, such as antipyrine, lidocaine and tranilast (TL) [4]. Transdermal drug delivery can permit a constant drug concentration in the dermal layers beneath the application site for a longer duration. However, there are no reports to evaluate a drug delivery system (DDS) to the ocular surface via the eyelid. Therefore, we attempted to design an ophthalmic formulation delivered to the lacrimal fluid through the eyelid.

There are recent reports of systems to deliver drugs into skin tissue, such as in situ gels. Iontophoresis [7], nanostructured lipid carriers [8,9], liposomes [10], dendrimers [11], phonophoresis [12], patches [13], micro-needles [14], and such systems can help overcome the low skin penetration [15]. However, the eyelid is easily stimulated by these systems, since the eyelid is the thinnest skin on the body and is highly sensitive to stimulus [16]. With these impediments, it is essential to search for an alternative ophthalmic delivery system while simultaneously improving drug absorption into the ocular tissues. On the other hand, we previously reported that formulations containing solid nanoparticles are safe, since these formulations do not involve toxic solvents in their preparation, such as surface-active agents, and show high penetration into skin tissues [17,18,19]. Therefore, we investigated whether a DDS based on solid nanoparticles can release drugs into the aqueous humor through the eyelid.

TL, N-(3,4-dimethoxycinnamoyl) anthramilic acid, reduces extracellular matrix production, matrix metalloproteinase-1 secretion, transforming growth factor beta 1 (TGF-β1) secretion, cell migration, chemotaxis and collagen synthesis [20,21,22]. For these reasons, oral formulations of TL are used clinically to treat atopic dermatitis [23] and allergic asthma [24], and TL is used as an anti-allergic conjunctivitis allergy drug in the ophthalmic field [25]. In addition, TL attenuates the development of posterior subcapsular opacification after cataract surgery [26] and the onset and recurrence of pterygium [27]. Moreover, TL prevents corneal haze after photorefractive keratectomy [28,29], and reduces cell adhesions after strabismus surgery [30]. Thus, TL is a safe and useful drug for treating allergic and inflammatory diseases in both the systemic and local field. In this study, we designed a sustained-release ophthalmic formulation based on TL nanoparticles for delivery through the eyelid.

## 2. Materials and Methods

### 2.1. Animals

Adult rabbits (male, weight 2.35 ± 0.39 kg, n = 18) were purchased from Shimizu Laboratory Supplies Co. Ltd. (Kyoto, Japan) The experimental protocol was approved by the Pharmacy Committee Guidelines for the Care and Use of Laboratory Animals in Kindai University (KAPS-25-003, 1 April 2013). All experiments were performed following the guidelines for ARVO. In this study, the treatment of TL formulations was performed at 14:00, and 0.75% TL formulations (0.3 g) were applied to the shaved upper eyelid skin (1.7 cm^2^).

### 2.2. Preparation of Ophthalmic Formulations Containing TL

Ophthalmic dispersions containing TL nanoparticles were prepared according to the previous reports [31,32]. TL powder (particle size 48.1 ± 3.1 μm) was obtained from Kissei Pharmaceutical Co., Ltd. (Nagano, Japan). Type SM-4 methylcellulose (MC) and 2-hydroxypropyl-β-cyclodextrin (HPβCD) were provided by Shin-Etsu Chemical Co., Ltd. (Tokyo, Japan) and Nihon Shokuhin Kako Co., Ltd. (Tokyo, Japan), respectively. The HPβCD and MC were selected to enhance the dispersion stability and crushing force, respectively [15,17,18,19]. Mixtures of these reagents (TL powder, MC and HPβCD) were dispersed in distilled water, and zirconia beads (0.1 mm in diameter) were added. The dispersions were crushed by a Bead Smash 12 (Wakenyaku Co., Ltd, Kyoto, Japan) at 5500 rpm for 30 min (1 min × 30 times). The milled dispersions were gelled with carboxypolymethylene (Carbopol^®^ 934, Serva, Heidelberg, Germany), and used as ophthalmic formulations containing TL nanoparticles (TL-NPs-Gel). In this study, formulations containing TL microparticles and dissolved TL (liquid TL) were also prepared and defined as TL-MPs-Gel and TL-liq-Gel (traditional ointment), respectively. The TL-MPs-Gel and TL-liq-Gel were prepared as follows. TL-MPs-Gel: TL, MC and HPβCD were dispersed in distilled water and gelled with carboxypolymethylene. TL-liq-Gel: TL, MC and HPβCD were dissolved in 0.8% DMSO and gelled with carboxypolymethylene (Carbopol^®^ 934). The ratios of TL, MC, and HPβCD in the TL formulations were 0.75%, 0.5% and 5%, respectively.

### 2.3. Analysis of Crystal Form

Samples were prepared as follows: mixtures of TL, MC and HPβCD were milled by the Bead Smash 12 as described above, and the milled TL was lyophilized, and used as samples. A powder X-ray diffraction (XRD) Mini Flex II (Rigaku Co., Tokyo, Japan) was used to analyze the crystal form, with the scanning rate, X-rays, and diffraction angles set to 10°/min, 30 kV and 15 mA, 5° to 90°, respectively.

### 2.4. Measurement of TL Levels

TL contents were measured by the HPLC method using an HPLC LC-20AT system (Shimadzu Corp. Kyoto, Japan). Ethyl p-hydroxybenzoate was used as an internal standard and the TL was added to 100 μL methanol containing ethyl p-hydroxybenzoate (3 mg/L). The mixture was injected into an Inertsil^®^ ODS-3 column (GL Science Co., Inc., Tokyo, Japan) at 35 °C, and detected at 230 nm. The mobile phase was 50 mM ammonium acetate and acetonitrile (80:20) at a flow rate of 0.25 mL/min.

### 2.5. Measurement of TL Particles

The TL formulations were diluted in distilled water and used as samples for measurement. The size dispersion of the TL-MPs-Gel was analyzed on a SALD-7100 (Shimadzu Corp., Kyoto, Japan) with the refractive index set at 1.60-0.10i. In addition, the size distribution and number of nanoparticles in the TL-NPs-Gel were measured using a NANOSIGHT LM10 (QuantumDesign Japan, Tokyo, Japan) with the viscosity, wavelength and measurement time set to 1.27 mPa⋅s, 405 nm and 60 s, respectively. Atomic force microscopic (AFM) images were provided by a SPM-9700 (Shimadzu Corp., Kyoto, Japan) according to previous report [33].

### 2.6. Ratios of Solid TL and Liquid TL in Formulations

The solid TL particles and the dissolved TL in the TL formulations were separated by centrifuging at 100,000 g in an Optima^TM^ MAX-XP Ultracentrifuge (Beckman coulter, Osaka, Japan). The collected TL particles were dissolved in methanol, and the ratios of solid TL particles and dissolved TL were measured by the HPLC method described above.

### 2.7. Photostability and Thermal Stability of TL Formulations

The TL formulations (0.3 g) were stored under 58 W/m^2^ florescent light (400–700 nm) at 25 °C for 24 h in the test for photostability. In the experiments for thermal stability, 0.3 g of TL formulations was kept at 60 °C for 10 weeks. The TL contents were measured by the HPLC method described above.

### 2.8. TL Release from TL Formulations

TL release from the TL formulations was evaluated as in previous studies using a Franz diffusion cell [15,17,18,19]. Briefly, a 220 nm-pore size MF™-MEMBRANE FILTER (Merck Millipore, Tokyo, Japan) was set into a Franz diffusion cell and 0.3 g of 0.75% TL formulation was applied to the filter. The reservoir chamber was filled with 10 mM phosphate (12.2 mL) and samples were collected from the reservoir chamber over time. The contents, nanoparticle number, and size distribution of TL in the samples were analyzed the HPLC and NANOSIGHT LM10 as described above.

### 2.9. TL Content in Lacrimal Fluid and Meibum of Rabbits

The eyelids of the rabbits were shaved and 0.3 g of TL formulations (0.75%) were applied to the 1.7 cm^2^ of shaved upper eyelids skin (outside), and samples of lacrimal fluid with or without meibum, or meibum only were collected. The Schirmer tear test strips were used to collect the lacrimal fluid in this study. Lacrimal fluid with meibum was collected from rabbits under the standard conditions by the insert the Schirmer tear test strips to the inside of eyelid. When lacrimal fluid without meibum was harvested, the outlet of the meibomian glands was covered with Schirmer tear test strips, a space was made between the eyelid and the ocular surface, and lacrimal fluid without meibum was collected by inserting the Schirmer tear test strips to inside of eyelid.

### 2.10. Statistical Analysis

Data are shown as the mean ± standard error (S.E.) with *P*-values less than 0.05 considered significant. Differences were analyzed by ANOVA followed by the Student’s *t*-test and Dunnett’s multiple comparisons.

## 3. Results

### 3.1. Design of An Ophthalmic Formulation Based on TL Solid Nanoparticles

**Figure 1:**
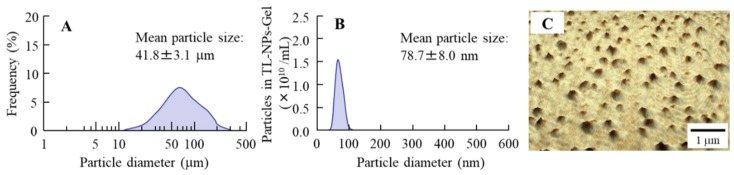
Size distribution of TL in TL-MPs-Gel and TL-NPs-Gel. (**A**) solid TL size in TL-MPs-Gel by SALD-7100. (**B**) solid TL size in TL-NPs-Gel by NANOSIGHT LM10. (**C**) AFM image of solid TL in TL-NPs-Gel. The TL nanoparticles were obtained by bead mill treatment at a particle size range of 50-100 nm.

**Figure 2:**
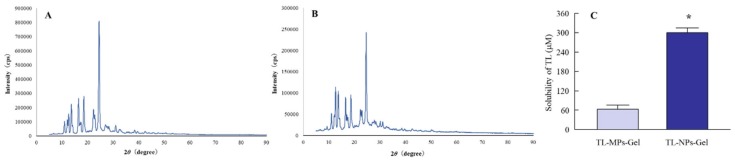
XRD pattern and solubility of TL in TL formulations. (**A**) and (**B**) XRD pattern of solid TL in TL-MPs-Gel (**A**) and TL-NPs-Gel (**B**) by the Mini Flex II. (**C**) solubilities of TL in TL-MPs-Gel and TL-NPs-Gel. N = 6. *P < 0.05, vs. TL-MPs-Gel. The XRD patterns of the crystalline materials show sharp peaks, and no differences with or without bead mill treatment. The ratio of dissolved TL in TL-NPs-Gel was 5.3-fold higher than that in TL-MPs-Gel.

**Figure 3:**
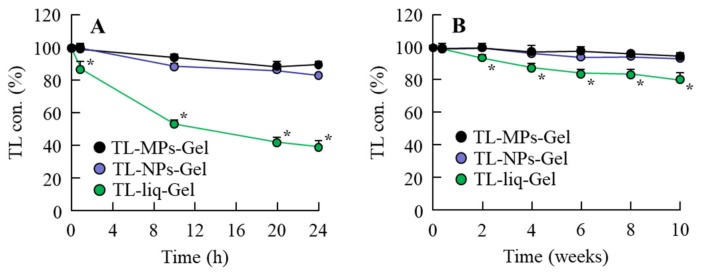
Photostability (**A**) and thermal stability (**B**) of TL in TL-NPs-Gel, TL-NPs-Gel and TL-liq-Gel. TL-MPs-Gel, formulation containing TL microparticles. TL-NPs-Gel, formulation containing TL nanoparticles. TL-liq-Gel, formulation containing dissolved TL (liquid TL). N = 5–8. *P < 0.05, vs. TL-NPs-Gel for each group. The photostability and thermal stability of TL-NPs-Gel was higher than those of TL-liq-Gel.

Figure 1 shows the change in particle size (Figure 1) produced by bead mill treatment, and Figure 2 shows the effects of bead mill treatment on the crystal structure and solubility of TL. The particle size of TL in TL-MPs-Gel was 10–300 µm, and this was decreased to 50–100 nm by bead mill treatment (TL-NPs-Gel; Figure 1). However, the crystal structures of the two preparations remained the same (Figure 2A,B). On the other hand, TL solubility was enhanced by bead mill treatment, with the solubility of TL-NPs-Gel 5.3-fold higher than that of TL-MPs-Gel. The ratio of solid to liquid TL was 98.7:1.3 for TL-NPs-Gel, most of the TL (98.7%) in TL-NPs-Gel is of the solid type (Figure 2C). Figure 3 shows the photochemical and thermal resistance in TL-NPs-Gel. The dissolved TL in TL-liq-Gel decomposed by irradiation under 58 W/m^2^ florescent light, leaving a TL content of 38.9% after 24 h of irradiation. In addition, the thermal resistance for the dissolved TL in TL-liq-Gel was low, with the TL content decreased to 80.2% after 24 h at 60 °C. Both the photochemical and thermal resistances of the solid TL in TL-MPs-Gel and TL-NPs-Gel were attenuated in comparison with TL-liq-Gel. the viscosity of TL-MPs, TL-NPs and TL-liq-Gel were 14.4 ± 0.8 Pa∙s, 14.9 ± 0.9 Pa∙s, 11.3 ± 0.8 Pa∙s (n = 5), respectively.

### 3.2. Sustained-Release of TL from TL-NPs-Gel 

**Figure 4:**
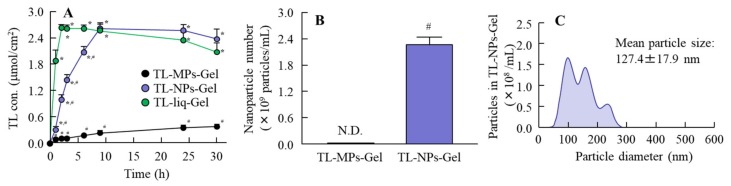
TL release from TL formulations through membranes. (**A**) TL release from TL formulations Table 220. nm pore membranes. (**B**) and (**C**) nanoparticle number (B) and size distribution (C) of TL in the reservoir chamber 24 h after the application of TL-NPs-Gel. TL-MPs-Gel, formulation containing TL microparticles. TL-NPs-Gel, formulation containing TL nanoparticles. TL-liq-Gel, formulation containing dissolved TL. n = 6. N.D., not detectable. *P < 0.05, vs. TL-MPs-Gel for each group. ^#^*P* < 0.05, vs. TL-liq-Gel for each group. The release of TL from TL-NPs-Gel reached a plateau 9 h after application; the TL was released in the form of nanoparticles.

Figure 4 shows the release of TL from TL-MPs-Gel, TL-NPs-Gel and TL-liq-Gel through a 220-nm pore size membrane in the Franz diffusion cell. The release of TL from TL-liq-Gel, which contains dissolved TL, took place earlier than the release from TL-MPs-Gel or TL-NPs-Gel, which contain mostly solid TL. The release of TL from TL-liq-Gel reached a plateau at 2 h, while TL release from TL-MPs-Gel and TL-NPs-Gel was slower, although the release of TL from TL-NPs-Gel was greater than that from TL-MPs-Gel. The release TL from TL-NPs-Gel reached a plateau 9 h after application. The TL levels at the plateau were similar for TL-NPs-Gel and TL-liq-Gel and were significantly higher than that for TL-MPs-Gel (Figure 4A). Moreover, the TL was migrated as nanoparticles from TL-NPs-Gel (Figure 4C), with 2.27 × 10^9^ nanoparticles detected in the reservoir chamber. 

### 3.3. Sustained-Release DDS for TL-NPs-Gel through the Eyelid

**Figure 5:**
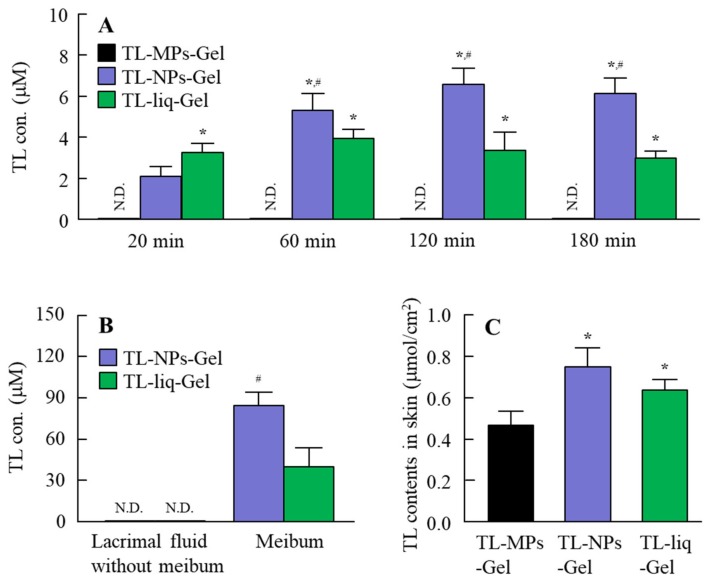
TL concentrations in the lacrimal fluid and meibum of rabbits treated with TL formulations. (**A**) TL concentration in the lacrimal fluid after treatment with TL formulations. (**B**) TL concentrations in the meibum and lacrimal fluid without meibum 180 min after treatment with TL formulations. (**C**) TL contents in the eyelid tissue 180 min after treatment with TL formulations. TL-MPs-Gel, formulation containing TL microparticles. TL-NPs-Gel, formulation containing TL nanoparticles. TL-liq-Gel, formulation containing dissolved TL. n = 5–7. N.D., not detectable. *P < 0.05, vs. TL-MPs-Gel for each group. ^#^*P* < 0.05, vs. TL-liq-Gel for each group. The TL in rabbits treated with TL-NPs-Gel was delivered to the lacrimal fluid through the meibomian glands.

Figure 5A shows the changes in TL levels in the lacrimal fluid of rabbits treated with TL formulations. Although TL levels were not detected in the lacrimal fluid of rabbits treated with TL-MPs-Gel, both TL-NPs-Gel and TL-liq-Gel delivered TL into the lacrimal fluid from the eyelid. The TL levels in the rabbits treated with TL-liq-Gel tended to be higher in comparison with TL-NPs-Gel at an early stage (20 min); however, the TL levels in rabbits treated with TL-NPs-Gel were significantly higher than in those treated with TL-liq-Gel at 60–180 min. Figure 5B shows the TL levels in the meibum 180 min after treatment with TL-NPs-Gel. The TL levels in the meibum of rabbits treated with TL-NPs-Gel were significantly higher than rabbits treated with TL-liq-Gel. In contrast with the results for meibum and lacrimal fluid with meibum, no TL was detected in the lacrimal fluid without meibum of rabbits treated with TL-NPs-Gel or TL-liq-Gel. In this study, we measured the number of TL nanoparticles in the meibum of rabbits treated with TL-NPs-Gel; however, only dissolved TL was delivered into the meibum (no TL nanoparticles were detected in the meibum). Figure 5C shows the TL contents in the eyelid tissue of rabbits treated with TL formulations. TL was delivered into the eyelid tissue from TL-MPs-Gel, TL-NPs-Gel and TL-liq-Gel, and the TL levels in eyelids of rabbits treated with TL-NPs-Gel were significantly higher than for rabbits treated with TL-MPs-Gel or TL-liq-Gel. After the application of TL formulation for 24 h, we observed the eyelid by the naked eye to investigate the stimulation of TL formulation, and the inflammation and redness was not observed in the administration of TL-MPs-Gel and TL-NPs-Gel. In addition, the TL in the lacrimal fluid of rabbit was detected 24 h after the treatment of TL-NPs-Gel.

## 4. Discussion

The residence time of traditional eye drops is short, and eye drops cannot be used when sleeping. Thus, the development of novel ophthalmic formulations to make sustained drug supplementation onto the ocular surface possible is highly expected. In this study, we designed a sustained-release ophthalmic formulation based on TL solid nanoparticles (TL-NPs-Gel) and investigated whether the application of TL-NPs-Gel to the eyelid can deliver TL into the lacrimal fluid.

In the preparation of solid nanoparticles, previous reports have shown the importance of the selection of additives [34]. We have also reported the method for preparing solid nanoparticles using bead mill treatment and showed that the addition of HPβCD and MC enhances the dispersion stability and crushing force, respectively [15,17,18,19]. In addition, our previous studies using indomethacin showed a hydrophilic gel base, such as carbopol, provides for a high drug release of solid nanoparticles from the gel [15,17,18,19]. Based on these findings, HPβCD, MC and carbopol were selected as components of TL-NPs-Gel in this study. The particle size of the TL in TL-NPs-Gel was 50 nm–100 nm (Figure 1), and the TL crystal structure was similar with and without bead mill treatment (Figure 2). In addition, the photostability of TL-NPs-Gel was higher than that of dissolved TL (Figure 3A). Kawabata et al. reported that the TL solution (liquid TL) is highly photodegradable, and that the photostability of 100–200 nm TL nanoparticles is higher than that of TL solution, since the degradation of TL was observed in the dissolved TL [35]. In this study, almost of all TL in the TL-MPs-Gel and TL-NPs-Gel was existed in the solid conditions (non-dissolved TL), and the TL-NPs-Gel showed high photochemical resistance under fluorescent light (400–700 nm) (Figure 3), supporting the previous report by Kawabata et al. [35]. Moreover, the thermal stability of TL-NPs-Gel was than that of TL solution (Figure 3). These results suggest that TL-NPs-Gel may be suitable for application as a gel or ointment, since the photostability and thermal stability of TL-NPs-Gel are higher than those of traditional formulations containing dissolved TL.

In this study, we investigated whether the TL in TL-NPs-Gel is released as nanoparticles, and used the membrane with 220 nm pore size, because it is expected to see no micro- and nanoparticles released from TL formulations in the reservoir chamber. There are differences in the release rates for the three TL formulations (Figure 4A). A rate in dissolved TL in TL-liq-Ge was faster than for TL-MPs-Gel and TL-NPs-Gel. Whereas, the release of TL from TL-NPs-Gel was higher than from TL-MPs-Gel. Next, we measured the number of nanoparticles in the reservoir chamber after treatment with TL formulations (Figure 4B,C). Although, no nanoparticles were detected in the reservoir chamber of Franz diffusion cell treated with TL-MPs-Gel, the TL nanoparticles was detected in the reservoir chamber of a Franz diffusion cell treated with TL-NPs-Gel (Figure 4B,C). These results show that the TL in TL-NPs-Gel is migrated continuously as solid nanoparticles. On the other hand, TL-NPs-Gel had heterogenous particle sizes after the release experiment. The 10-mM phosphate was filled in the reservoir chamber, and the released TL nanoparticles and additives (HPβCD and MC) were diluted by the 10 mM phosphate. This dilution of additives may cause the decrease in the particle stability of TL-NPs-Gel.

**Figure 6:**
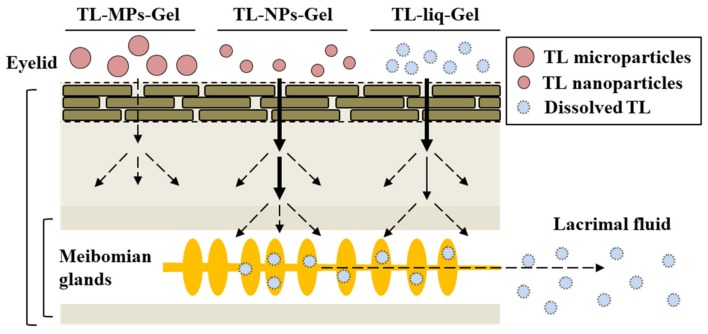
Ophthalmic drug delivery routes through the eyelid of TL formulations.

After the experiment examining drug release from gel formulations, the changes in the TL concentration in the lacrimal fluid were demonstrated (Figure 5). At 180 min after treatment, the TL contents in the conjunctiva of rabbits treated with TL-MPs-Gel, TL-NPs-Gel and TL-liq-Gel were 0.47, 0.75 and 0.63 μmol/cm^2^, respectively (Figure 5C); however, no TL was detected in the lacrimal fluid after treatment with TL-MPs-Gel. In contrast to the results for TL-MPs-Gel, TL was delivered into the lacrimal fluid after treatment with TL-NPs-Gel and TL-liq-Gel. The TL concentration in the lacrimal fluid of rabbits treated with TL-NPs-Gel was higher than in rabbits treated TL-liq-Gel, and the TL release profile for TL-NPs-Gel was sustained for 180 min (Figure 5A). In this study, we also demonstrated the permeation pathway to the lacrimal fluid from the eyelid surface. For both TL-NPs-Gel and TL-liq-Gel, no TL delivery to other side through the skin was detected. The eyelid tissue contains large sebaceous glands, the meibomian glands, which secrete meibum, an oily substance that prevents evaporation into the lacrimal fluid [36]. Therefore, the TL concentration in the meibum was measured to clarify the penetration pathway from the eyelid to the lacrimal fluid (Figure 5B). TL was detected in the meibum of rabbits treated with TL-NPs-Gel and TL-liq-Gel, with the TL concentration in the meibum of rabbits treated with TL-NPs-Gel was higher than in rabbits treated with TL-liq-Gel. The ratios of the TL levels in the lacrimal fluid and meibum were similar rabbits treated with both formulations (Figure 5A,B). Meibum is a sebaceous solution, and the sebaceous solutions show a high binding affinity for hydrophobic drugs in general. Therefore, it was hypothesized that TL-NPs-Gel penetrates the skin, where a part of the penetrated TL shifts to the meibomian glands, where the TL is dissolved since no TL nanoparticles were detected in the meibum. After that, the TL is delivered to the lacrimal fluid in the meibum (Figure 6). Our previous studies using rebamipide showed that the endocytosis was related to the eyelid skin penetration of solid nanoparticles [37]. Therefore, the endocytosis pathway may also be related the penetration in TL-NPs-Gel. On the other hand, we did not investigate whether the TL solid nanoparticles in TL-NPs-Gel is penetrated via stratum corneum or hair follicle. Moreover, we do not have data for TL condition at the deep region of eyelids. In addition, it is important to perform in vitro and in vivo studies to prove TL nanoparticles effectiveness and biocompatibility. Further studies are needed to confirm the penetration mechanism, effectiveness and biocompatibility of TL solid nanoparticles.

## 5. Conclusions

An ophthalmic formulation based on TL solid nanoparticles (TL-NPs-Gel) provides sustained TL supplementation to the lacrimal fluid, probably through the meibomian glands, to the ocular surface from the eyelid. These findings concerning a sustained-release ophthalmic formulation and the route of penetration through the meibomian glands provide significant information to develop ophthalmic formulations. Further studies are needed to develop a TL nano-delivery system through the eyelid, and it is important to clarify the mechanism of the trans-eyelid penetration of TL-NPs-Gel.
